# Different guidelines for pre-exposure prophylaxis (PrEP) eligibility estimate HIV risk differently: an incidence study in a cohort of HIV-negative men who have sex with men, Portugal, 2014–2018

**DOI:** 10.2807/1560-7917.ES.2020.25.28.1900636

**Published:** 2020-07-16

**Authors:** Paula Meireles, Michael Plankey, Miguel Rocha, João Brito, Luís Mendão, Henrique Barros

**Affiliations:** 1 EPIUnit–Instituto de Saúde Pública, Universidade do Porto, Porto, Portugal; 2 Georgetown University Medical Center, Georgetown University, Washington DC, United States; 3 GAT–Grupo de Ativistas em Tratamentos, Lisboa, Portugal; 4 Coalition PLUS Community-Based Research Laboratory, Patin, France; 5 Departamento de Ciências da Saúde Pública e Forenses e Educação Médica, Faculdade de Medicina, Universidade do Porto, Porto, Portugal

**Keywords:** HIV, incidence, pre-exposure prophylaxis, men who have sex with men, eligibility

## Abstract

**Introduction:**

Guidelines for pre-exposure prophylaxis (PrEP) provide criteria to identify individuals at higher risk of HIV infection. We compared the ability to predict HIV seroconversion of four guidelines: the World Health Organization (WHO), the United States Public Health Service and Centers for Disease Control and Prevention (US CDC), the European AIDS Clinical Society (EACS) and the Portuguese National Health Service (PNHS).

**Aim:**

We aimed to measure the association between guideline-specific eligibility and HIV seroconversion.

**Methods:**

We studied 1,254 participants from the Lisbon Cohort of men who have sex with men with at least two evaluations between March 2014 and March 2018, corresponding to 1,724.54 person-years (PY) of follow-up. We calculated incidence rates (IR) according to each guideline eligibility definition and incident rate ratios (IRR) to test the association between eligibility at baseline and HIV seroconversion.

**Results:**

We found 28 incident cases (IR: 1.62/100 PY; 95% confidence interval (CI) 1.12–2.35). Guidelines’ sensitivity varied from 60.7% (EACS) to 85.7% (PNHS) and specificity varied from 31.8% (US CDC) to 51.5% (EACS). IR was highest among those defined as eligible by the PNHS guideline (2.46/100 PY; IRR = 4.61; 95% CI: 1.60–13.27) and lowest for the WHO guideline (1.89/100 PY; IRR = 1.52; 95% CI: 0.69–3.35).

**Conclusions:**

Being identified as eligible for PrEP was associated with a higher risk of infection. The magnitude of risk varied according to the guideline used. However, the number of HIV infections identified among ineligible participants highlights the potential for missing people who need PrEP.

## Introduction

The current prevention armamentarium for human immunodeficiency virus (HIV) has several effective strategies, such as treatment as prevention, medical male circumcision, condom use, behavioural change, pre-exposure prophylaxis (PrEP) and post-exposure prophylaxis (PEP). When used in combination, these strategies have the potential to reverse the HIV epidemic [1-3]. One key aspect of a public health approach to combination prevention is the ability to identify those at higher risk correctly [4]. While some strategies, such as condom use, are intended to reach the highest number of individuals, other strategies, such as PrEP, primarily target individuals at higher risk to maximise cost-effectiveness [5]. Several screening tools and guidelines exist that help healthcare providers identify high-risk individuals based on HIV predictors [6-9]. However, they were associated with moderate discrimination in predicting incident HIV infections [10].

PrEP is the use of antiretroviral therapy, usually tenofovir disoproxil fumarate and emtricitabine, to prevent HIV in adolescents and adults at high risk of infection, including men who have sex with men (MSM) [11-13]. It was first approved by the United States (US) Food and Drug Administration in 2012, then, in 2016, by the European Medicines Agency (EMA) and is now available in several countries, including Portugal. In Portugal, PrEP is available through the Portuguese National Health Service (PNHS), fully reimbursed, since February 2018.

PrEP has been shown to be very effective in reducing incidence of HIV infections. The pooled relative reduction in randomised clinical trials conducted among MSM was estimated at 77% but highly correlated with adherence [10]. Clinical guidelines were designed to help healthcare professionals in the provision of PrEP by defining the eligibility criteria to identify those at higher risk of infection.

Guidelines recommend the use of PrEP for sexually active individuals without acute or established HIV infection who are at high risk of acquiring HIV. Their specific criteria include known predictors of HIV seroconversion such as condomless anal intercourse, having an HIV-positive sexual partner who is not virally suppressed and a diagnosis of a sexually transmitted infection. However, only some published guidelines include the number of partners, substance use or history of PEP. Using different guidelines results in different proportions of eligibility in the same population, as we have previously shown [14]. Further, we hypothesise that this may also result in different ability to predict HIV seroconversion.

HIV incidence is expected to be higher among those eligible for PrEP. However, some studies reported an unsatisfactory sensitivity of the guidelines from the US Public Health Service and Centers for Disease Control and Prevention (CDC) [15-17]. 

We wanted to provide real-world evidence of the ability of different international guidelines to predict HIV seroconversion, using data from a cohort of HIV-negative MSM testing at a community-based voluntary HIV counselling and testing (CBVCT) centre in Lisbon, Portugal. Thus, we compared HIV incidence according to eligibility for PrEP defined by (i) the World Health Organization (WHO), (ii) the US CDC, (iii) the European AIDS Clinical Society (EACS) and (iv) the PNHS, and we measured the association between guideline-specific eligibility and HIV seroconversion.

## Methods

The Lisbon Cohort of MSM is an ongoing prospective cohort study conducted at a CBVCT in Lisbon, Portugal (CheckpointLX). A description of the cohort is provided elsewhere [18,19]. In brief, the Lisbon Cohort of MSM is an open, non-interval cohort of men 18 years or older who report having sex with men, present for an HIV test at CheckpointLX and have a negative HIV test result at recruitment. All individuals meeting these criteria are invited to enter the cohort by CheckpointLX’s peer community health workers (CHW) at their first visit. Follow-ups occur when participants come for another HIV test; no fixed time between visits is defined. At each visit, a structured questionnaire is administered using an online form, and a rapid HIV test is performed by a trained CheckpointLX peer CHW. Pre-test and post-test counseling are offered at every visit in an opt-out strategy. Recruitment started in April 2011, but data reported in this study refer to the period from March 2014 to March 2018.

### Participants 

For this study, we considered the 3,713 adult MSM who presented for a first test at CheckpointLX between March 2014 and March 2018 and accepted to complete a baseline questionnaire. Of them, 148 (4.0%) had an HIV-reactive result and were not eligible for follow-up. Among the remaining 3,565, 1,347 came for at least one follow-up visit. Of those, 93 were excluded from the analysis because they reported use of PrEP (n = 46), could not be classified as eligible or ineligible by one or more guidelines at baseline (n = 46) or for both reasons (n = 1). Thus, we analysed 1,254 participants, corresponding to a total follow-up of 1,724.54 person-years (PY), with a median number of two visits and a median time of 7 months and 18 days between visits.

### Study instruments and variables

PrEP eligibility was defined according to four different guidelines: (i) module 1 of the WHO’s Implementation Tool for Preexposure Prophylaxis of HIV Infection [20], (ii) the CDC/US Public Health Service’s Preexposure Prophylaxis for the Prevention of HIV Infection in the United States–2017 Update [21], (iii) the EACS Guidelines Version 9 [22] and (iv) the Portuguese clinical guidelines from the National Health Service [23]. The criteria were matched with the behavioural information collected in the Lisbon Cohort of MSM baseline questionnaire (available from the authors on request) and were operationally defined as described in Table 1. A more detailed description is available elsewhere [14]. Information regarding the 12 months before the baseline was used, except for the EACS criterion regarding the use of PEP, for which lifetime information was used. We were not able to compute the EACS criterion related to chemsex, defined as ‘sexual intercourse under the influence of recreational drugs taken predominantly intravenously immediately before and/or during sexual contacts’ [22] because we do not collect information about the predominant mode of drug administration. We were also not able to compute the PNHS criterion related to ‘persons in situations of social vulnerability that may expose them to unprotected sexual intercourse with individuals at high risk of acquiring HIV infection’ [23] because we do not have an objective measure of social vulnerability. Participants were defined as eligible according to a given guideline when they met the respective criteria. We excluded those for whom information was incomplete because it was missing or because they had answered ‘rather not say’ or ‘do not know’. We also collected information on age, country of birth (categorised in world regions except for Portugal and Brazil where numbers were high), educational level, sexual identity, history of a previous HIV test and reasons for the index test (a list of 12 reasons is provided of which more than one reason can be chosen; for this analysis we have categorised hierarchically the reasons in terms of self-perception of risk as follows: related to symptoms, related to risk exposure and not related to symptoms or risk exposure).

**Table 1:** Operational definition of each eligibility criterion in the WHO, US CDC, EACS and PNHS guidelines for pre-exposure prophylaxis (PrEP) eligibility

Guideline and criteria for eligibility	Operational definition of eligibility^a^
WHO criteria (2017) [20]	
1. Vaginal or anal sexual intercourse without a condom with more than one partner, or	Any anal intercourse with steady or occasional partners without a condom AND more than one sexual partner
2. A recent history (in the last 6 months) of an STI by laboratory testing or self-report or syndromic STI treatment, or	Self-report of syphilis, chlamydia, lymphogranuloma venereum, gonorrhoea, trichomoniasis, genital herpes, condyloma or genital warts, or other STI diagnosis
3. PEP for sexual exposure in the past 6 months, or	Use of PEP
4. Sexual partner with HIV who is not taking suppressive ART	Anal intercourse with steady partner AND having at least one HIV-positive steady partner AND having at least one HIV-positive partner who is not taking treatment OR whose HIV status is not known OR who had detectable or unknown viral load
US CDC criteria (2017) [21]	
1. Any male sex partners in the past 6 months, and	Any anal intercourse with steady or occasional partners
2. Not in a monogamous partnership with a recently tested, HIV-negative man, and any of the following	except men reporting only one HIV-negative male steady partner and no occasional partners
3. Any anal sex without condoms (receptive or insertive) in the past 6 months, or	Any anal intercourse with steady or occasional partners without a condom
4. Any STI diagnosed or reported in the past 6 months, or	Self-report of syphilis, chlamydia, lymphogranuloma venereum, gonorrhoea, trichomoniasis, genital herpes, condyloma or genital warts, or other STI diagnosis
5. In an ongoing sexual relationship with an HIV-positive male partner	Anal intercourse with steady partner AND having at least one HIV-positive steady partner
EACS criteria (2017) [22]	
1. Inconsistent condom use with casual partners, or	Any anal intercourse with occasional partners without a condom
2. Recent STI, or	Self-report of syphilis, chlamydia, lymphogranuloma venereum, gonorrhoea, trichomoniasis, genital herpes, condyloma or genital warts, or other STI diagnosis
3. Use of PEP, or	Use of PEP (lifetime)
4. Inconsistent condom use with HIV-positive partners who are not receiving treatment	Anal intercourse with steady partner AND having at least one HIV-positive steady partner AND having at least one HIV-positive partner who is not taking treatment AND any anal intercourse with steady partners without a condom
PNHS criteria (2018) [23]	
1. Persons who have had condomless intercourse in the past 6 months and sexual partners with unknown HIV status, or	Any anal intercourse with steady or occasional partners without a condom AND having at least one sexual partner for whom the HIV status is unknown
2. People who refer to the use of psychoactive substances during sexual intercourse, or	Used at least one psychoactive substance during intercourse, including cannabis, heroin, cocaine, ecstasy, amphetamines, poppers, LSD, ketamine, GHB, methadone, substances sold at smart shop, methamphetamines, mephedrone or other
3. Persons who have had condomless intercourse in the past 6 months and had an STI diagnosis, or	Any anal intercourse with steady or occasional partners without a condom AND self-report of syphilis, chlamydia, lymphogranuloma venereum, gonorrhoea, trichomoniasis, genital herpes, condyloma or genital warts, or other STI diagnosis
4. Persons who have had condomless intercourse in the past 6 months and used PEP for HIV, or	Any anal intercourse with steady or occasional partners without a condom AND use of PEP
5. People whose partner is infected with HIV, without medical care or ART, or without virological suppression and who do not use condoms consistently, or	Anal intercourse with steady partner AND having at least one HIV-positive steady partner AND having at least one HIV-positive partner who is not taking treatment or whose HIV status is not known OR who had detectable or unknown viral load AND any anal intercourse with steady or occasional partners without a condom
6. People who engage in sexual intercourse to obtain money or goods or illicit substances and do not use condoms consistently	People who report having received money, goods, or drugs in exchange for sexual intercourse AND any anal intercourse with steady or occasional partners without a condom

ART: antiretroviral therapy; EACS: European AIDS Clinical Society; GHB: gamma-hydroxybutyric acid; HIV: human immunodeficiency virus; LSD: lysergic acid diethylamide; PEP: post-exposure prophylaxis; PNHS: Portuguese National Health Service; STI: sexually transmitted infection; US CDC: United States Centers for Disease Control and Prevention; WHO: World Health Organization.

^a^ Information regarding the 12 months before the baseline was used, except for the EACS criterion regarding the use of PEP, for which lifetime information was used.

HIV testing was performed using rapid tests. A third-generation test (Alere Determine HIV-1/2, Alere Medical Co Ltd, Chiba, Japan) was used until October 2016 and again since November 2017. A fourth-generation test (Alere Determine, HIV-1/2 Ag/Ab Combo, Alere Medical Co Ltd, Chiba, Japan) was used from October 2016 to October 2017. In case of a reactive test result, a referral was offered to the public hospital HIV/infectious diseases clinic most convenient to the participant, where a confirmatory test would be performed. Results from the confirmation of infection were not provided to CheckpointLX; however, two participants informed that their result did not confirm. Therefore, seroconversions were defined as having a reactive result, unless the participant informed CheckpointLX that his infection did not confirm.

### Statistical analysis

We described the participants using counts and proportions and computed incidence rates (IR) for participants defined as eligible and ineligible at baseline according to each guideline and by each criterion. Time at risk was computed as the period between recruitment and the most recent follow-up visit. For those MSM who seroconverted, we subtracted half of the period between the last HIV-negative test result and the HIV-positive test result. To measure the magnitude of the association between being eligible for PrEP at baseline and acquiring HIV during follow-up, we computed crude incidence rate ratios (IRR) and respective 95% confidence intervals (CI) using generalised linear models with Poisson regression, with the default log link and offset in the variable time at risk. Statistical analysis was computed with SPSS for Windows, version 23.0 (SPSS Inc, Chicago, IL). To evaluate a guideline’s performance in identifying participants who seroconverted, we computed the sensitivity (i.e. the proportion of eligible individuals among participants who seroconverted) and the specificity (i.e. the proportion of ineligible participants among those who did not seroconvert). We also computed the number needed to treat (NNT) to prevent one HIV infection among eligible individuals under three scenarios: (i) a relative reduction of 97% as reported in the open-label extension of the ANRS IPERGAY study [24], (ii) a relative reduction of 86% as in the ANRS IPERGAY trial and PROUD study [12,13] and (iii) a relative reduction of 77% as in a meta-analysis of randomised clinical trials among MSM [10]. We used this relative reduction to calculate the expected IR had PrEP been given to eligible individuals. Then the NNT could be computed as the reciprocal of the IR difference.

### Ethical statement

All participants provided written informed consent before inclusion, and the study protocol was approved by the ethics committee of São João Hospital Center and Medical School, University of Porto (ID 104/12).

## Results

A description of the overall sample and by HIV status at the end of follow-up is presented in Table 2. At baseline, the 1,254 participants had a median age of 27.1 years (25th–75th percentiles: 23.0–35.3), 965 (77.0%) were born in Portugal, and foreign-born individuals were mostly from Brazil (n = 122, 9.7%) and other European countries (n = 111; 8.9%). Participants who seroconverted reported more frequently than those who remained negative to have been born in Brazil (6/28; 21.4% vs 116/1,226; 9.5%) or an African country (2/28; 7.1% vs 30/1,226; 2.4%). More than 80% of participants self-identified as gay and more than half had a higher education degree (among participants that seroconverted less than half had higher education; 13/28). The most reported reasons for testing were related to the perception of being exposed to a risk situation for HIV (66.6%); this proportion was higher (71.4%) among participants who seroconverted. Having a previous HIV test was reported by 958 participants (76.4%).

**Table 2:** Description at baseline of the overall sample and by HIV status at the end of follow-up, Portugal, 2014–2018 (n = 1,254)

Characteristics	Participants n = 1,254		HIV status at the end of follow-up					
			HIV-negative n = 1,226			HIV-positive n = 28		
**Age (years)**								
Mean (standard deviation)	30.0 (9.34)		30.0 (9.39)			29.6 (6.99)		
Median (25th–75th percentile)	27.1 (23.0–35.3)		27.1 (22.9 - 35.4)			28.5 (23.4–34.0)		
Range	18.0–69.0		18.0–69.1			19.9–43.5		
	**n**	**%**	**n**		**%**	**n**		**%**
**Country/region of origin**								
Portugal	965	77.0	948	77.3		17	60.7	
Brazil	122	9.7	116	9.5		6	21.4	
Other European country	111	8.9	108	8.8		3	10.7	
African country	32	2.6	30	2.4		2	7.1	
Other American country	16	1.3	16	1.3		0	0.0	
Asia / Middle east / Oceania	8	0.6	8	0.7		0	0.0	
**Educational level**								
Basic education or less	50	4.0	50	4.1		0	0.0	
Secondary education	428	34.1	414	33.8		14	50.0	
Professional training	40	3.2	39	3.2		1	3.6	
Post-secondary education	14	1.1	14	1.1		0	0.0	
Bachelor	452	36.0	442	36.1		10	35.7	
Master or doctoral degree	269	21.5	266	21.7		3	10.7	
Rather not say	1	0.1	1	0.1		0	0.0	
**Sexual identity**								
Gay	1,037	82.7	1,014	82.7		23	82.1	
Bisexual	177	14.1	172	14.0		5	17.9	
Heterosexual	12	1.0	12	1.0		0	0.0	
Other/does not use a term/does not know	27	2.2	27	2.2		0	0.0	
Rather not say	1	0.1	1	0.1		0	0.0	
**Previous HIV testing**								
No	296	23.6	290	23.7		6	21.4	
Yes	958	76.4	936	76.3		22	78.6	
**Reason for the index test**								
Reasons related to symptoms^a^	76	6.1	74	6.0		2	7.1	
Reasons related to risk exposure^b^	835	66.6	815	66.5		20	71.4	
Reasons not related to symptoms or risk exposure^c^	333	26.6	327	26.7		6	21.4	
Missing	10	0.8	10	0.8		0	0.0	
**Eligible for PrEP**								
**World Health Organization**								
Ineligible	489	39.0	480	39.2^d^		9	32.1	
Eligible	765	61.0	746	60.8		19	67.9^e^	
**United States Centers for Disease Control and Prevention**								
Ineligible	396	31.6	390	31.8^d^		6	21.4	
Eligible	858	68.4	836	68.2		22	78.6^e^	
**European AIDS Clinical Society**								
Ineligible	642	51.2	631	51.5^d^		11	39.3	
Eligible	612	48.8	595	48.5		17	60.7^e^	
**Portuguese National Health Service**								
Ineligible	495	39.5	491	40.0^d^		4	14.3	
Eligible	759	60.5	735	60.0		24	85.7^e^	

HIV: human immunodeficiency virus; PrEP: pre-exposure prophylaxis.

^a^ Participants reported ‘Symptoms/medical indication’.

^b^ Participants did not report ‘symptoms/medical indication’ and reported at least one of the following reasons: anonymous partner notification’, ‘partner was diagnosed with HIV/disclosed HIV status’, ‘window period in the previous test’, ‘condom failure’, ‘perception of recent exposure to HIV’, or ‘perception of exposure to HIV more than 3 months’.

^c^ Participants did not report ‘symptoms/medical indication’ and did not report any of the reasons coded as related to risk exposure and reported at least one of the following reasons: ‘asked by a sexual partner’, ‘before discontinuing using the condom with my partner’, ‘beginning of a new relationship’, ‘end of relationship with my usual partner’, or ‘to know health status/routine’.

^d^ These values represent the specificity of the guidelines.

^e^ These values represent the sensitivity of the guidelines.

At baseline, 61.0% of participants were eligible for PrEP according to the WHO guidelines, 68.4% according to the US CDC guidelines, 48.8% according to the EACS guidelines and 60.5% according to the PNHS guidelines. Among those who acquired HIV during follow-up, the proportion of eligible participants (sensitivity) varied from 60.7% (17/28) according to the EACS guidelines, to 85.7% (24/28) according to the PNHS guidelines. The proportion of ineligible participants among those who remained HIV-negative (specificity) varied from 31.8% according to the US CDC guidelines to 51.5% according to the EACS guidelines (Table 2).

Table 3 presents the results concerning HIV incidence and its association with eligibility for PrEP. During follow-up, there were 28 incident infections in a total of 1,724.54 PY at risk, yielding an incidence rate of 1.62 (95% CI: 1.12–2.35) per 100 PY. Most seroconversions were observed among those defined as eligible for PrEP according to the PNHS guidelines, corresponding to an HIV incidence of 2.46 per 100 PY (95% CI: 1.65–3.67). The HIV incidence per 100 PY among ineligible participants was also lowest according to the PNHS guidelines (0.53; 95% CI: 0.20–1.42).

**Table 3:** Association between HIV incidence and eligibility for PrEP according to the WHO, US CDC, EACS and PNHS guidelines, Portugal, 2014–2018 (n = 1,254)

	HIV cases	Person-years	IR per 100 person-years (95% CI)	IRR (95% CI)
Overall	28	1,724.54	1.62 (1.12–2.35)	Not applicable
**Eligibility for PrEP at baseline**				
**World Health Organization (2017)**				
Ineligible	9	720.95	1.25 (0.65–2.40)	Reference
Eligible	19	1,003.59	1.89 (1.21–2.97)	1.52 (0.69–3.35)
**United States Centers for Disease Control and Prevention (2017)**				
Ineligible	6	601.66	1.00 (0.45–2.22)	Reference
Eligible	22	1,122.87	1.96 (1.29–2.98)	1.96 (0.80–4.85)
**European AIDS Clinical Society (2017)**				
Ineligible	11	928.01	1.19 (0.66–2.14)	Reference
Eligible	17	796.53	2.13 (1.33–3.43)	1.80 (0.84–3.84)
**Portuguese National Health Service (2018)**				
Ineligible	4	748.85	0.53 (0.20–1.42)	Reference
Eligible	24	975.69	2.46 (1.65–3.67)	4.61 (1.60–13.27)

CI: confidence interval; EACS: European AIDS Clinical Society; HIV: human immunodeficiency virus; IR: incidence rate; IRR: incidence rate ratio; PNHS: Portuguese National Health Service; PrEP: pre-exposure prophylaxis; SD: Standard deviation; US CDC: United States Centers for Disease Control and Prevention; WHO: World Health Organization.

A strong association (IRR = 4.61; 95% CI: 1.60–13.27) was found between being eligible according to the PNHS guidelines at baseline and HIV seroconversion. Being eligible according to the other guidelines was associated with a 52% increase in HIV incidence in the case of the WHO guidelines (IRR = 1.52; 95% CI: 0.69–3.35), 80% in the case of the EACS guidelines (IRR = 1.80; 95% CI: 0.84–3.84) and 96% in the case of the US CDC guidelines (IRR = 1.96; 95% CI: 0.80–4.85) (Table 3). However, for all but the PNHS guidelines, the CI overlapped 1.

Table 4 shows the participants’ distribution by each guideline criterion: the most frequently met criteria were those related to condom use. HIV incidence was highest among those meeting the EACS criterion of ‘inconsistent condom use with casual partners’ (IR = 2.37; 95% CI: 1.45–3.87) and the PNHS criterion of ‘persons who have had condomless sex in the past 6 months and sexual partners with unknown HIV status’ (IR = 2.76; 95% CI: 1.74–4.38) and the criterion of ‘people who refer to use of psychoactive substances during sexual intercourse’ (IR = 2.49; 95% CI: 1.41–4.38). These criteria also presented the highest lower bound of the confidence interval.

**Table 4:** HIV incidence by criteria for eligibility for PrEP according to the WHO, US CDC, EACS and PNHS guidelines, Portugal, 2014–2018 (n = 1,254)

Guideline and criteria for eligibility^a^	Participants meeting the criterion		HIV cases	Person-years	IR per 100 person-years (95% CI)
	n	%			
**World Health Organization (2017)**					
1. Vaginal or anal sexual intercourse without a condom with more than one partner	713	56.9	19	937.47	2.03 (1.29–3.18)
2. A recent history (in the last 6 months) of an STI by laboratory testing or self-report or syndromic STI treatment	116	9.3	3	149.16	2.01 (0.65–6.24)
3. PEP for sexual exposure in the past 6 months	30	2.4	0	32.88	0.00 (0.00–11.22)
4. Sexual partner with HIV who is not taking suppressive ART	35	2.8	0	40.82	0.00 (0.00–9.04)
**United States Centers for Disease Control and Prevention (2017)**					
1. Any male sex partners in the past 6 months	1,214	96.8	28	1,660.12	1.69 (1.16–2.44)
2. Not in a monogamous partnership with a recently tested, HIV-negative man	1,190	94.9	28	1,622.00	1.73 (1.19–2.50)
3. Any anal sex without condoms (receptive or insertive) in the past 6 months	862	68.7	22	1,146.50	1.92 (1.26–2.91)
4. Any STI diagnosed or reported in the past 6 months	116	9.3	3	149.16	2.01 (0.65–6.24)
5. Is in an ongoing sexual relationship with an HIV-positive male partner	71	5.7	0	78.41	0.00 (0.00–4.70)
**European AIDS Clinical Society (2017)**					
1. Inconsistent condom use with casual partners	517	41.2	16	674.86	2.37 (1.45–3.87)
2. Recent STI	116	9.3	3	149.16	2.01 (0.65–6.24)
3. Use of PEP	61	4.9	0	59.27	0.00 (0.00–6.22)
4. Inconsistent condom use with HIV-positive partners who are not receiving treatment	15	1.2	0	12.97	0.00 (0.00–28.44)
**Portuguese National Health Service (2018)**					
1. Persons who have had condomless sex in the past 6 months and sexual partners with unknown HIV status	524	41.8	18	652.80	2.76 (1.74–4.38)
2. People who engage in sexual intercourse to obtain money, goods or illicit substances and do not use condoms consistently	16	1.3	0	19.53	0.00 (0.00–18.89)
3. Persons who have had condomless sex in the past 6 months and had an STI diagnosis	89	7.1	3	112.36	2.67 (0.86–8.28)
4. Persons who have had condomless sex in the past 6 months and used PEP for HIV	25	2.0	0	24.62	1.65 (1.14–2.39)
5. People whose partner is infected with HIV without medical care or ART or without virological suppression and do not use condoms consistently	17	1.4	0	15.08	0.00 (0.00–24.46)
6. People who refer to the use of psychoactive substances during sexual intercourse	368	29.3	12	481.97	2.49 (1.41–4.38)

ART: antiretroviral therapy; IR: incidence rate; EACS: European AIDS Clinical Society; HIV: human immunodeficiency virus; PEP: post-exposure prophylaxis; PNHS: Portuguese National Health Service; STI: sexually transmitted infection; US CDC: United States Centers for Disease Control and Prevention; WHO: World Health Organization.

^a^ As defined in the guidelines.

Table 5 presents the estimates for the number of people who need to take PrEP for 1 year in order to avert one HIV infection, assuming different relative reductions. The lowest estimates varied from 42 to 53, with the PNHS guidelines having the lowest values across all scenarios.

**Table 5:** Estimates for the expected incidence rate and number needed to treat for 1 year under different scenarios of relative reduction and eligibility defined according to the different guidelines, Portugal, 2014–2018 (n = 1,254)

Study	ANRS IPERGAY (open-label extension) [**25**]		PROUD study [**13**] and ANRS IPERGAY trial [**12**]		Meta-analysis of RCTs among MSM [**10**]	
Relative reduction	97%		86%		77%	
Guideline used	Expected IR/100 PY	NNT	Expected IR/100 PY	NNT	Expected IR/100 PY	NNT
WHO (2017)	0.057	54	0.265	61	0.435	69
US CDC (2017)	0.059	53	0.274	59	0.451	66
EACS (2017)	0.064	48	0.299	54	0.491	61
PNHS (2018)	0.074	42	0.344	47	0.566	53

ANRS: Agence Nationale de Recherches sur le Sida et les Hépatites Virales; EACS: European AIDS Clinical Society; IPERGAY: Intervention Préventive de l’Exposition aux Risques avec et pour les Gays; IR: incidence rate; MSM: men who have sex with men; NNT: number needed to treat; PNHS: Portuguese National Health Service; PROUD: Pre-exposure Option for Reducing HIV in the UK; PY: person-years; RCT: randomised clinical trial; US CDC: United States Centers for Disease Control and Prevention; WHO: World Health Organization.

## Discussion

Using these four guidelines for PrEP, the proportion of incident cases that would be eligible for PrEP at baseline varied from 60% to more than 85%, meaning that, in the worst scenario of PrEP eligibility identification and relative reduction, at least half of the infections could have been avoided. Overall, HIV incidence was 1.62 per 100 PY; this was higher among participants defined as eligible for PrEP, independently of the guideline used, varying from 1.89 per 100 PY when the WHO guidelines were used to 2.46 per 100 PY when the PNHS guidelines were used.

The PNHS guidelines were able to identify the highest number of seroconverters (85.7%) and showed the strongest association with seroconversion (IRR = 4.61; 95% CI: 1.60–13.27). Being eligible according to the other guidelines was also associated with an increased HIV incidence, but the magnitude of those associations was lower, and all CI included 1. Even when approximately the same number of eligible participants at baseline resulted from different guidelines, their discriminating ability was different, leading to a range of the NNT varying from 42 to 69. These estimates of the NNT are higher than the one estimated by the PROUD study conducted at sexual health clinics in England between 2012 and 2014 [13], but the baseline HIV incidence rates are very different, being much lower in this Portuguese setting. We chose to use these three scenarios to be able to provide estimates under a range of relative reductions that are mainly dependent on adherence to treatment.

These differences among guidelines can be due to the differences in the eligibility criteria and their relevance or ability to capture the drivers of HIV transmission. The predictors of HIV seroconversion in this cohort have been previously described and were similar to those found in other MSM cohorts [18,25-28]. All these aspects were generally included in the guidelines. However, condomless anal sex with a steady partner, for instance, independent of HIV status, is not included in the WHO and EACS guidelines and can lead to missing those MSM to whom the steady partner had not yet disclosed his HIV status (whether previously diagnosed or not). Reportedly not knowing the HIV status of the sexual partners with whom condomless sex occurred and having used psychoactive substances during sexual intercourse were included as criteria only in the PNHS guidelines, which may explain their strong association with seroconversion as both criteria had two of the highest incidence rates in our cohort. These parameters should receive consideration in defining or updating guidelines for PrEP use among MSM.

A study conducted in Madrid, Spain among MSM and transgender women (97.8% were cisgender men) recently diagnosed as having HIV, found that 86.6% had an indication for PrEP according to the national AIDS study group guidelines, a sensitivity similar to the one showed by the best operating guidelines [29]. Yet, our ability to make comparisons with previous studies is limited because most studies evaluated guidelines’ ability to identify HIV seroconversion in the US using the US CDC guidelines.

Our results show that the eligibility criteria were able to identify a large number of MSM who, in fact, seroconverted. However, having as much as 39% of seroconversions among participants defined as ineligible at baseline should be highlighted. This suggests that people who do not fill the eligibility criteria may still need PrEP. However, we must acknowledge that changes in the eligibility status may have occurred during follow-up, which, as we have previously shown, influences seroconversion risk [18]. Nevertheless, it is important to highlight that there was a substantial number, varying according to the guideline used, of HIV seroconversions among ineligible participants. It was previously shown that the US CDC criteria failed to identify a considerable proportion of individuals at risk for HIV [9,16,17], and the same was observed in this study and for the other guidelines. Previous research also suggests that people not meeting the eligibility criteria but, for instance, requesting PrEP may be at risk of HIV seroconversion [30,31, 32]. In line with this, the Australasian guidelines state that clinicians may deem a person at risk and recommend or consider PrEP even though the candidate does not meet their criteria [33]. Also, changes to improve guidelines’ performance in identifying HIV seroconverters among specific populations of MSM have been suggested; these were to include psychosocial components as well as network or other population-level factors besides individual-level factors [9,16,34]. All these factors highlight the tension between what guidelines recommend, what clinicians think is best and what individuals want.

Our study has limitations that need to be acknowledged. Firstly, exposure ascertainment can lead to misclassifications for two main reasons: (i) the variables collected in questionnaires of the cohort are not exactly phrased as the criteria and (ii) our analysis was grounded in behavioural risk and not in clinical eligibility, with the exception of the HIV antibody determination; therefore, there was no clinical information to assess any contraindication for PrEP, which may overestimate the expected advantages. The timing of exposure ascertainment should also be discussed. We opted to use baseline information for two main reasons: (i) we wanted to guarantee a longitudinal design and make sure that the ascertainment of eligibility preceded the seroconversion, and (ii) we wanted to be closer to a scenario in which MSM may not be at an imminent risk of HIV acquisition but seeking for PrEP (as they did for HIV testing) and are classified as eligible or not. This approach, however, does not account for changes in eligibility during follow-up and, in some cases, may be a distant predictor. Nevertheless, ca 50% had only two visits and a median time between visits of 7.5 months. Secondly, taking into consideration the number of seroconversions observed and the related effect on precision, estimates need to be cautiously considered. We were not able to determine eligibility according to the PNHS guidelines for the period from inception to March 2014, which was possible for the other three guidelines. When they were evaluated using the entire period, the direction and magnitude of the associations for the WHO, US CDC, and EACS guidelines were similar to the results presented here (data provided in Supplementary Table 1). Thirdly, external validity might be limited if the drivers of the epidemic are different in other settings and time periods. Information bias caused by a large number of losses to follow-up may also influence the association between eligibility and seroconversion. Although participants with follow-up visits presented different sociodemographic characteristics at baseline from those with no follow-up in terms of country of birth and educational level, there were no differences in the mean age, sexual orientation, previous HIV test, reasons for the index test and eligibility for PrEP, except for the EACS guidelines (Supplementary Table 2). Finally, another source of bias to our estimates may be related to social desirability and recall of information. We aimed to reduce these by the peer-based approach provided by CheckpointLX. Nevertheless, we cannot exclude the possibility of under-reporting of risk behaviours.

## Conclusion

The observed number of new HIV cases and the incidence rate were highest among those defined as being eligible for PrEP according to the PNHS guidelines, suggesting their adequacy identifying MSM at high risk of HIV infection. Still, all guidelines were able to identify those at higher risk. Nonetheless, the substantial number of HIV infections among ineligible participants should highlight the potential of missing people in need of PrEP. This study shows that further work is needed to improve the performance of guidelines or alternative approaches to assess candidacy for PrEP.

## Supplementary Data

135000 Supplement
