# Supplementary material for: Different guidelines for pre-exposure prophylaxis (PrEP) eligibility estimate HIV risk differently: an incidence study in a cohort of HIV-negative men who have sex with men, Portugal, 2014–2018
Source: Euro Surveill. 2020 Jul 16;25(28):1900636. doi: 10.2807/1560-7917.ES.2020.25.28.1900636 (PMC7376846; doi:10.2807/1560-7917.ES.2020.25.28.1900636)
Supplement: Supplement [file 19-00636_Supplement_MEIRELES.pdf]

Supplementary table 1. Association between HIV incidence and eligibility for PrEP according to the WHO, US CDC, and EACS guidelines, Portugal, 2014-2018 (n=2,398).

Disclaimer: This supplementary material is hosted by *Eurosurveillance* as supporting information alongside the article entitled 'Different guidelines for pre-exposure prophylaxis (PrEP) eligibility estimate HIV risk differently: an incidence study in a cohort of HIV-negative men who have sex with men, Portugal, 2014-2018', on behalf of the authors, who remain responsible for the accuracy and appropriateness of the content. The same standards for ethics, copyright, attributions and permissions as for the article apply. Supplements are not edited by *Eurosurveillance* and the journal is not responsible for the maintenance of any links or email addresses provided therein.

	HIV cases	Person-years	IR per 100 person-years (95% CI)	IRR (95% CI)
**Overall**	97	5,257.75	1.84 (1.51-2.25)	Not applicable
**Eligibility for PrEP at baseline**				
**World Health Organization (2017**				
Ineligible	32	2,326.67	1.38 (0.97-1.94)	Reference
Eligible	65	2,931.08	2.22 (1.74-2.83)	1.61 (1.06-2.46)
**United States Centers for Disease Control and Prevention (2017)**				
Ineligible	22	1,847.26	1.19 (0.78-1.81)	Reference
Eligible	75	3,410.49	2.20 (1.75-2.76)	1.85 (1.15-2.97)
**European AIDS Clinical Society (2017)**				
Ineligible	49	2,982.77	1.64 (1.24-2.17)	Reference
Eligible	48	2,274.98	2.11 (1.59-2.80)	1.28 (0.86-1.91)

CI: confidence interval; EACS: European AIDS Clinical Society; HIV: human immunodeficiency virus; IR: incidence rate; IRR: incidence rate ratio; PrEP: preexposure prophylaxis; US CDC: United States, Centers for Disease Control and Prevention; WHO: World Health Organization.

Supplementary table 2: Description at baseline of participants with and without follow-up visits among those who with complete information about the eligibility status at baseline, Portugal, 2014-2018.

Disclaimer: This supplementary material is hosted by *Eurosurveillance* as supporting information alongside the article entitled 'Different guidelines for pre-exposure prophylaxis (PrEP) eligibility estimate HIV risk differently: an incidence study in a cohort of HIV-negative men who have sex with men, Portugal, 2014-2018', on behalf of the authors, who remain responsible for the accuracy and appropriateness of the content. The same standards for ethics, copyright, attributions and permissions as for the article apply. Supplements are not edited by *Eurosurveillance* and the journal is not responsible for the maintenance of any links or email addresses provided therein.

Characteristics	Participants without follow-up N=2,095		Participants with follow-up N=1,254		p-value
**Age (years)**					
Mean (standard deviation)	30.6 (9.48)		30.0 (9.34)		0.063 [a]
Median, 25th-75th percentile	28.7 (23.5-35.6)		27.1 (23.0-35.3)		0.012 [b]
Range	16.2-74.4		18.0-69.0		n.a.
	n	%	n	%	
**Country/region of origin**					
Portugal	1500	71.6	965	77.0	0.001 [c]
Brazil	271	12.9	122	9.7	
Other European country	185	8.8	111	8.9	
African country	56	2.7	32	2.6	
Other American country	48	2.3	16	1.3	
Asia/Middle east/Oceania	34	1.6	8	0.6	
**Educational level**					
Basic education or less	119	5.7	50	4.0	0.031 [c]
Secondary education	656	31.4	428	34.2	
Professional training	51	2.4	40	3.2	
Postsecondary	26	1.2	14	1.1	
Bachelor	831	39.7	452	36.1	
Master or doctoral	409	19.6	269	21.5	
Rather not say	2		1		
**Sexual identity**					
Gay	1729	82.7	1037	82.8	0.557 [c]
Bisexual	286	13.7	177	14.1	
Heterosexual	32	1.5	12	1.0	
Other/does not use a term/does not know	44	2.1	27	2.2	
Rather not say	4		1		
**Previous HIV testing**					
No	493	23.5	296	23.6	0.996 [c]
Yes	1602	76.5	958	76.4	
**Reason for the index test**					
Reasons related to symptoms [d]	145	7.0	76	6.1	0.215 [c]
Reasons related to risk exposure [e]	1339	64.2	835	67.1	
Reasons not related to symptoms or risk exposure [f]	602	28.9	333	26.8	
Missing	9		10		
**Eligible for PrEP, no**					
**World Health Organization**					
Ineligible	884	4.2	489	39.0	0.074 [c]
Eligible	1211	57.8	765	61.0	
**United States Centers for Disease Control and Prevention**					
Ineligible	679	32.4	396	31.6	0.645 [c]
Eligible	1416	67.6	858	68.4	
**European AIDS Clinical Society**					
Ineligible	1154	55.1	642	51.2	0.032 [c]
Eligible	941	44.9	612	48.8	

Portuguese National Health Service					
Ineligible	824	39.3	495	39.5	0.870[c]
Eligible	1271	60.7	759	60.5	

[a] p-value for the t-test for independent samples

[b] p-value for the Mann-Whitney test

[c] p-value for the chi-square test

[d] Participants reported 'Symptoms/medical indication.'

[e] Participants did not report 'Symptoms/medical indication' and reported at least 1 of the following reasons: 'Anonymous partner notification,' 'Partner was diagnosed with HIV/disclosed HIV status,' 'Window period in the previous test,' 'Condom failure,' 'Perception of recent exposure to HIV,' or 'Perception of exposure to HIV more than 3 months.'

[f] Participants did not report 'Symptoms/medical indication' and did not report any of the reasons coded as related to risk exposure and reported at least 1 of the following reasons: 'Asked by a sexual partner,' 'Before discontinuing using the condom with my partner,' 'Beginning of a new relationship,' 'End of relationship with my usual partner,' or 'To know health status/routine.'

HIV: human immunodeficiency virus; n.a.: not applicable; PrEP: preexposure prophylaxis.
